# Successful Invasions of Short Internally Deleted Elements (SIDEs) and Its Partner CR1 in Lepidoptera Insects

**DOI:** 10.1093/gbe/evz174

**Published:** 2019-08-06

**Authors:** Ping-Lan Wang, Andrea Luchetti, Angelo Alberto Ruggieri, Xiao-Min Xiong, Min-Rui-Xuan Xu, Xiao-Gu Zhang, Hua-Hao Zhang

**Affiliations:** 1 College of Pharmacy and Life Science, Jiujiang University, China; 2 Dipartimento di Scienze Biologiche, Geologiche e Ambientali, Università di Bologna, Italy; 3 Clinical Medical College, Jiujiang University, China

**Keywords:** chicken repeat 1 (CR1), transposable elements evolutionary dynamics, long interspersed element (LINE), Lepidoptera, short internally deleted element (SIDE), vertical inheritance

## Abstract

Although DNA transposons often generated internal deleted derivatives such as miniature inverted-repeat transposable elements, short internally deleted elements (SIDEs) derived from nonlong terminal-repeat retrotransposons are rare. Here, we found a novel SIDE, named *Persaeus*, that originated from the chicken repeat 1 (CR1) retrotransposon *Zenon* and it has been found widespread in Lepidoptera insects. Our findings suggested that *Persaeus* and the partner *Zenon* have experienced a transposition burst in their host genomes and the copy number of *Persaeus* and *Zenon* in assayed genomes are significantly correlated. Accordingly, the activity though age analysis indicated that the replication wave of *Persaeus* coincided with that of *Zenon*. Phylogenetic analyses suggested that *Persaeus* may have evolved at least four times independently, and that it has been vertically transferred into its host genomes. Together, our results provide new insights into the evolution dynamics of SIDEs and its partner non-LTRs.

## Introduction

The eukaryotic genome is composed by a wide diversity of transposable elements (TE), some autonomous (i.e., coding for the enzymatic machinery necessary for replication and reintegration) and some others nonautonomous (i.e., dependent on autonomous-encoded enzymes for replication and reintegration) (Chénais et al. 2012). Among nonautonomous elements, there are the short interspersed elements (SINEs) that are nucleotide (nt) sequence made by different modules (head, body, and tail) with different origins (Luchetti and Mantovani 2013). Other kind nonautonomous elements are internally deleted copies of autonomous elements. Miniature inverted-repeat transposable elements (Feschotte and Pritham 2007) and terminal-repeat retrotransposons in miniature (Gao et al. 2016) are widespread elements, derived from internal deletions of autonomous DNA transposons and long-terminal-repeat retrotransposons (LTR), respectively. On the contrary, short internally deleted elements (SIDEs) originated from non-LTR elements seems to be rare, being only found in fruit flies, in the mosquito *Anopheles gambiae* and in the protozoan *Trypanosoma brucei* (Kimmel et al. 1987; Biedler and Tu 2003; Eickbush and Eickbush 2012).

Chicken repeat 1 (CR1) elements are non-LTRs, long interspersed elements (LINEs) and were the first TE found in the chicken genome about three decades ago (Stumph et al. 1981, 1984). CR1 replicates through a “copy-and-paste” mechanism and, usually, shows two open reading frames (ORFs) coding for a Gag-like protein, which has a zinc finger motif, and a Pol-like protein, which has endonuclease and reverse transcriptase (RT) domains (Burch et al. 1993; Haas et al. 1997; Kajikawa et al. 1997). Compared with L1 LINE, 5′-UTR of CR1 elements are more frequently truncated, which imply a lower processivity of its transcription (Hillier et al. 2004).

CR1 elements are the most abundant TE families in the genomes of birds (Hillier et al. 2004; Warren et al. 2010), crocodilians (Green et al. 2014), snakes (Castoe 2013), and turtles (Shaffer et al. 2013) and are composed by a great diversity that existed from the era of the common ancestor of amniotes (Suh et al. 2014). CR1 elements are also the only active TEs throughout the evolution of birds and, thus, have been widely served as genetic markers (Kaiser et al. 2006; Haddrath and Baker 2012; Liu et al. 2012; Baker et al. 2014). However, the evolutionary history and dynamics of CR1 elements in insects remain largely unknown. So far, CR1 have been found in a few insects, namely some flies (Kapitonov and Jurka 2003; Thompson et al. 2009), the mosquito *A. gambiae* (Biedler and Tu 2003) and some Lepidoptera (butterflies and moths) species (Novikova et al. 2007), where it may show even only a single ORF encoding endonuclease and RT domains. To our best knowledge, there was only one documented example of SIDEs originated from CR1 elements (Biedler and Tu 2003).

In this study, we report on the finding of a novel SIDE, derived from a CR1 element, isolated from the genome of Lepidoptera insects. Obtained results suggested that this SIDE as well as its partner *Zenon* have been highly active during the evolution of some Lepidoptera superfamilies and that the SIDE may have evolved multiple times, independently. Moreover, although widespread among Lepidoptera, our results suggest a vertical inheritance at least at lower taxonomic level. Overall, we concluded that SIDE and *Zenon* reported here might provide a good system to study the dynamics of emergence of SIDEs and their interaction with the partner LINE.

## Materials and Methods

### Animal Materials

Dazao, a strain of the silkworm *B. mori*, was obtained from the State Key Laboratory of Silkworm Genome Biology (China). *Antheraea pernyi* and *A. yamamai* were collected from Heilongjiang province (China) and Changbai Mountain (Jilin province, China), respectively. *Rhodnius prolixus* was kindly provided by Dr Ricardo Nascimento Araujo (Laboratório de Fisiologia de Insetos Hematófagos, Brazil). *Samia insularis*, *Samia luzonica*, *Samia cynthia ricini*, *Amathuxidia amythaon* and *Caligo eurilochus* was purchased from Shanghai Qiuyu Biotechnology Co., Ltd (China). Then, we extracted their total DNAs using TIANamp Genomic DNA Kit (TIANGEN).

### PCR, Cloning, and Sequencing

We designed a pair of specific primers (Forward: 5′-GAG CCG ATT GTT GAA GCG GAA AAA G-3′; Reverse: 5′-TGG CCT TGA TAG CGT TGT TCA AAA T-3′) of *Garfield_BM* (Zhang et al. 2014) using its internal sequence to determine its distribution in some insects. PCR was performed with an initial denaturation step of 4 min at 95 °C followed by 30 cycles of 40 s at 95 °C, 40 s at 58 °C, and 2 m at 72 °C. Then, purified PCR products were cloned into PMD-19 cloning vector (TaKaRa). One or two random clones of each species were selected and sequenced.

### Sequence Analyses

Two SIDEs search strategies have been implemented. In the first, SIDEs were found in published Lepidoptera genomes by BLASTing the *A. pernyi* SIDE sequence with the *blastn* algorithm and e-value >10^−10^. In the second, Zenon was first found by means of *tblastn* algorithm (e-value >10^−5^) of BLAST search using the RT domain as query sequence; once characterized the Zenon nt sequence, the 5′ and the 3′ end where manually joined and used to BLAST search as described above. When the search gave significant positive hits, the first full-length 50 hits were used to build a majority rule consensus sequence. This consensus sequence was, then, used to perform an exhaustive search on relative genomes using the same BLAST search parameters. All positive hits were used to build a new, final SIDE consensus sequence for each genome. In addition to genomes scan, also the nonredundant nt, ESTs, and TSA NCBI databases (accessed on May 2019) were probed with all consensus sequences in order to find further SIDE copies.

**Fig. 1.**
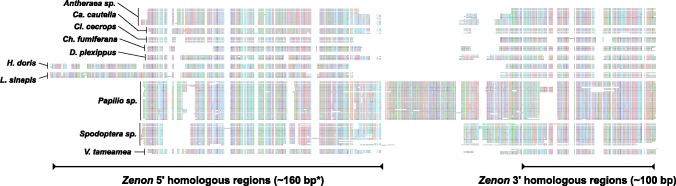
—Schematic view of *Persaeus* sequences (five copies per species) with indication of *Zenon* homologous regions. Approximate length of homologous regions is also reported (* *H. doris* and *L. sinapis* homologous 5′ end is ∼250 bp).

The search for partner LINE *Zenon* elements was performed following the same procedure. The only exception was that in some instances no full-length copies were retrieved: When possible, the complete *Zenon* sequence was reconstructed by manually aligning BLAST hit regions and recognizing the element borders. In some instance, we were unable to reconstruct the full-length sequence, so that those elements were no further considered. All obtained consensus sequences were, then, validate by checking the presence of ORF translating in an RT domain. SIDE and partner LINE copy number determination and activity through age analysis have been carried out on genomes using RepeatMasker v. 4.0 (Smit et al. 2013–2015). However, because the homology between the SIDE and the LINE could determine that consensus sequences mask each other copies we decided to exclude fragments long <160 bp (250 bp in the case of *Leptidea sinapis*): This allowed to recover fragment unambiguously belonging to the SIDE or to the LINE (fig. 1). Moreover, to further refine the copy number estimation, adjacent fragments were merged into single hits using the script Onecodetofindthemall.pl (Bailly-Bechet et al. 2014).

In the activity through age analysis, the relative repeat abundances are plotted against the Jukes–Cantor genetic divergence (which takes into account also multiple substitutions) of each repeat copy versus the consensus sequence of its family. The less divergent copies are the most recently transposed, and the most divergent are those whose replication occurred far in the past. 

Phylogenetic analyses were carried out through maximum likelihood and Bayesian inference. Maximum likelihood was performed with RAxML v.8 (Stamatakis 2014) using the GTR+G substitution model (*Parsaeus* and *Zenon* nt data sets) or rtREV+G model (*Zenon* RT amino acid data set) and 100 rapid bootstrap replicates. Bayesian inference was done using MrBayes v. 3.2 (Ronquist et al. 2012), with the same models as above: 2 independent runs searched for 10^6^ generation and trees were sampled every 100. Convergence of the two runs was reached when the average variance of split frequencies <0.01 and Potential Scale Reduction Factor approached ∼1.0. The final Bayesian consensus tree was obtained after a conservative *burnin*=25%.

## Results

### Identification of a Novel SIDE and Its Partner LINE

A survey on the distribution of a *Chapaev* transposon named *Garfield* identified in our previous study (Zhang et al. 2014) in some insects was performed using polymerase chain reaction (PCR): One PCR amplification band obtained from the Chinese tussar moth *Antheraea pernyi* was ∼350 bp longer than the expected band size (supplementary fig. S1, Supplementary Material online). After cloning and sequencing, we found that *Garfield* from the Chinese tussar moth had an additional insertion of 352 bp. This insertion exhibited a poly-(A) 3′ end and seemed to be flanked by a (T)_6_ target site duplication (supplementary fig. S2, Supplementary Material online). A homology search in the Repbase Update database (Jurka 2000; last accessed October 2018) evidenced that the full length of this insertion shared ∼70% of nt sequence identity with CR1 autonomous elements, named *Zenon*, from two lepidopteran species: *Heliconius melpomene* and *Papilio xuthus*. More in detail, a sequence comparison indicated that homologous regions are at the 5′ end, overlapping the 5′-UTR and the beginning of the *Zenon* ORF, and at the 3′ end, encompassing the end of the ORF and the whole 3′-UTR of the *Zenon* elements including the poly-(A) tail (supplementary fig. S3, Supplementary Material online). The insertion does not show any homology with those of tRNA, 5S rRNA, or 7S rRNA genes and lacks an RNA pol III promoter, which are two major characteristics that distinguish SINEs from other nonautonomous transposons (Luchetti and Mantovani 2013). Therefore, this suggested that the insertion found in the *Garfield* element from the Chinese tussar moth is, actually, an SIDE derived from an internal deletion of the *Zenon* element. This novel SIDE has been named *Persaeus*, as he was the favorite disciple of the Greek philosopher Zenon of Citium.

### Taxonomic Distribution of *Persaeus* and *Zenon*

We investigated the distribution of the SIDE *Persaeus* and its partner LINE *Zenon* in other genomes available at the National Center for Biotechnology Information (NCBI; last accessed June 2019), including the nonredundant nt, expressed sequence tags (EST), and transcriptome sequences assembly (TSA). We found that *Persaeus* was present in the genome of 21 Lepidoptera species belonging to the Bombycoidea, Pyraloidea, Papilionoidea, Tortricoidea, Noctuoidea superfamilies (table 1). The copy number ranged from 12 in *Vanessa tameamea* (Papilionoidea) to 115,283 in *Calycopis cecrops* (Papilionoidea), covering up to the 3.68% of the genome (supplementary table S1, Supplementary Material online). We found *Zenon* in the same also present in additional six species, two of which belonging to further superfamilies: Gelechioidea and Hesperioidea. The copy number varied from 245 in *Danaus chrysippus* (Papilionoidea) to 40,029 in in *Leptidea sinapis* (Papilionoidea; table 1); they cover up to the 3.45% of the genome of *Leptidea sinapis* (supplementary table S1, Supplementary Material online). No positive hits were found outside Lepidoptera in any peered database.

**Table 1:** Detailed Information of *Persaeus* SIDEs and the Associated LINE *Zenon* in This Study

Species	Taxonomy (Superfamily)	*Persaeus*	*Persaeus* Copy Number	*Persaeus* Length (bp)	*Zenon*	*Zenon* Copy Number	*Zenon* Length (bp)	Database/Genome Assembly Acc. no.
*Antheraea assama*	Bombycoidea	✓		271	✓		3,315	TSA
*A. pernyi*	Bombycoidea	✓		317	✓		3,316	TSA
*A. yamamai*	Bombycoidea	✓		302				TSA
*Bombyx mandarina*	Bombycoidea				✓	5,963	3,534	GCA_003987935.1
*Cadra cautella*	Pyraloidea	✓		316				TSA
*Calephelis nemesis*	Papilionoidea				✓	8,938	3,461	GCA_002245505.1
*Calycopis cecrops*	Papilionoidea	✓	115,283	263	✓	12,324	3,386	GCA_001625245.1
*Choristoneura fumiferana*	Tortricoidea	✓		274				EST
*Danaus chrysippus*	Papilionoidea				✓	245	3,352	GCA_004959915.1
*Danaus plexippus*	Papilionoidea	✓	3,019	304				GCA_000235995.2
*Heliconius melpomene*	Papilionoidea				✓	1,461	3,392	GCA_000313835.2
*H. numata*	Papilionoidea				✓	1,115	3,396	GCA_900068715.1
*H. doris*	Papilionoidea	✓	821	341				GCA_900068325.1
*Hyposmocoma kahamanoa*	Gelechioidea				✓	6,419	3,064	GCA_003589595.1
*Leptidea sinapis*	Papilionoidea	✓	158	366	✓	40,029	3,366	GCA_900199415.1
*Megathymus ursus*	Hesperioidea				✓	14,562	3,741	GCA_003671415.1
*Papilio dardanus*	Papilionoidea	✓		382				nt
*P. glaucus*	Papilionoidea	✓	37,447	386	✓	5,516	3,340	GCA_000931545.1
*P. machaon*	Papilionoidea	✓	15,080	386	✓	1,502	3,282	GCA_001298355.1
*P. memnon*	Papilionoidea	✓	8,993	381	✓	752	3,300	GCA_003118335.3
*P. polytes*	Papilionoidea	✓	14,479	382	✓	999	3,270	GCA_000836215.1
*P. xuthus*	Papilionoidea	✓	12,723	384	✓	1,549	3,339	GCA_000836235.1
*P. zelicaon*	Papilionoidea	✓		386				TSA
*Spodoptera exigua*	Noctuoidea	✓		302				TSA
*S. frugiperda*	Noctuoidea	✓	19,971	313	✓	1,350	3,315	GCA_002213285.1
*S. littoralis*	Noctuoidea	✓		316				TSA
*S. litura*	Noctuoidea	✓	30,212	317	✓	2,736	3,274	GCA_002706865.1
*Vanessa tameamea*	Papilionoidea	✓	12	281	✓	5,456	3,364	GCF_002938995.1

Overall, we got *Persaeus*/*Zenon* pair (i.e., the two elements from the same genome) from 12 species. On the other hand, for nine species we only got *Persaeus* and for six species we only found *Zenon* (these do not include *H. melpomene* and *Bombyx mori* for which the LINE was already known): Although in most cases this could be related to the databases where the species have been assayed, that could be limited and containing only repeat fragments such as nt, EST, or TSA databases, the exclusive presence of *Persaeus* or *Zenon* has been observed also in complete genomes (table 1).

### Structure and Phylogenetic Analysis of *Persaeus* Elements

We collected a sample of 5, full-length copies of the *Persaeus* element from the 21 lepidopteran species in order to compare the sequence structure and variability. The resulting alignment can be partitioned in three main blocks: The 5′ and 3′ *Zenon* homologous regions and a variable central region (fig. 1). The two *Zenon* homologous regions showed a similar average nt identity of 66.0% and 67.6%, respectively. Moreover, a visual inspection of the alignment revealed a remarkable structural diversity among species, whereas repeats from congeneric species showed a more consistent structural pattern (fig. 1). The central variable region was found containing nt fragments that appear taxon-specific and whose homology among taxa do not seem obvious (fig. 1). 

**Fig. 2.**
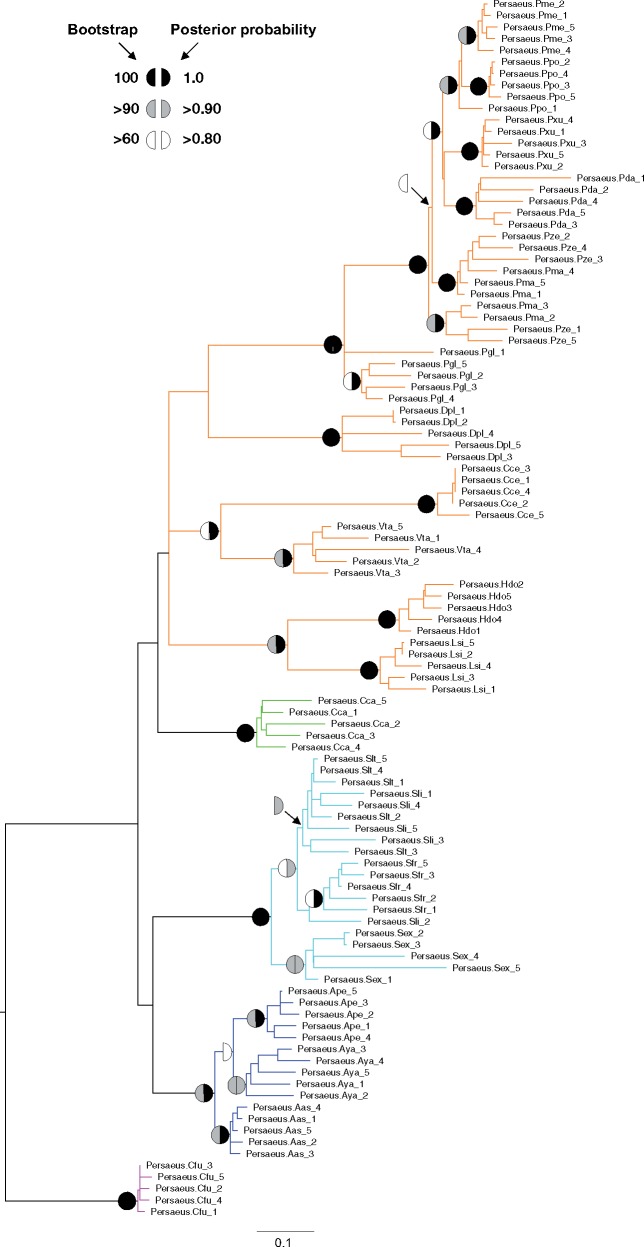
—Phylogenetic analysis *Persaeus* elements (five copies per species). Symbols at nodes represent maximum likelihood bootstrap/Bayesian posterior probability node support, as reported in the upper left legend. Branch color codes are indicative of the lepidopteran superfamily, as follow: Orange, Papilionoidea; blue, Bombycoidea; cyan, Noctuoidea; green, Pyraloidea; magenta, Tortricoidea. Each element has been labelled by a suffix indicating the pertaining species: Aya, *Antheraea yamamai*; Ape, *A. pernyi*; Aas, *A. assama*; Cca, *Cadra cautella*; Cce, *Calycopis cecrops*; Cfu, *Choristoneura fumiferana*; Dpl, *D. plexippus*; Hdo, *H. doris*; Lsi, *Leptidea sinapis*; Pda, *Papilio dardanus*; Pgl, *P. glaucus*; Pma, *P. machaon*; Pme, *P. memnon*; Ppo, *P. polytes*; Pxu, *P. xuthus*; Pze, *P. zelicaon*; Sex, *Spodoptera exigua*; Sfr, *S. frugiperda*; Slt, *S. littoralis*; Sli, *S. litura*; Vta, *Vanessa tameamea*.

Maximum likelihood and Bayesian inference phylogenetic trees obtained using the 105 *Persaeus* copies resulted in two completely overlapping topologies: These are mostly unresolved at deep nodes but show higher support at the most recent nodes (fig. 2). Overall, SIDE sequences form species-specific clusters with the exception of repeats from *Papilio machaon*/*P. zelicaon* and *Spodoptera litura*/*S. littoralis* species pairs that are intermingled within their respective cluster (fig. 2). At genus level, SIDEs from *Antheraea* spp., *Papilio* spp., and *Spodoptera* spp. are included in the three, clearly monophyletic clades. At higher taxonomic level, Papilionoidea (the only superfamily for which more than one genus is available) are included in a single cluster, although not supported by maximum likelihood bootstrap or Bayesian posterior probabilities. Here, beside *Papilio* spp., two other species pairs are included in supported monophyletic groups: *C. cecrops*/*Vanessa tameamea* and *Heliconius dori*/*Leptidea sinapis* (fig. 2).

### Phylogenetic Analysis of *Zenon* Elements

We obtained full-length *Zenon* elements from 18 lepidopteran species. The RT protein domain was then used for phylogenetic analysis of newly isolated elements together with *Zenon* obtained from RepBase Update, Zenon-1_Hmel, Zenon-2_Hmel, Zenon-3_Hmel from *H. melpomene* and Zenon_BM from *B. mori*, and closely related CR1 elements from *H. melpomene* genome. Both maximum likelihood and Bayesian inference were congruent and are presented in supplementary figure S4, Supplementary Material online. The *Zenon* clade appeared monophyletic, although weakly supported; all *Zenon* elements for which a *Persaeus* SIDE has been isolated fell in the same supported cluster but intermingling with other *Zenon* elements obtained from genomes lacking the SIDE. As observed for *Persaeus* phylogeny, there are no clear relationships at superfamily taxonomic level but elements from congeneric species are consistently clustered together. The only exceptions are *B. mori* and *B. mandarina* elements that are paraphyletic with the remaining *Zenon* elements. Moreover, *Heliconius* spp. and *L. sinapis* elements are assembled in a supported cluster (supplementary fig. S4, Supplementary Material online).

### Structural and Evolutionary Relationship between *Persaeus* and *Zenon*

In all SIDE/LINE pairs it is well clear the homology at the 5′ and 3′ end regions (supplementary dataset S1, Supplementary Material online). The nt identity between 5′ ends of each pair ranges from 71.0% in *Papilio glaucus* to 98.9% in *V. tameamea*, whereas the identity between 3′ ends ranges from 60.4% in *Papilio polytes* to 99.2% in *V. tameamea* (supplementary table S2, Supplementary Material online). In the *Heliconius* genomes, we only got *Persaeus* from *H. doris*, where *Zenon* was not observed; on the other hand, *Zenon* was found in the congeneric *H. melpomene* and *H. numata*. Despite they are present in different genomes, the identity between the homologous regions spans from 94.2% (*Persaeus H. doris* vs. Zenon-1_Hmel 3′ end) to 96.5% (*Persaeus H. doris* vs. Zenon-1_Hmel 5′ end) (supplementary table S2, Supplementary Material online). This holds also for *Danaus* spp. genomes, where *Persaeus* was found in *D. plexippus* but not *D. chrysippus* and vice versa for *Zenon* (table 1). Though, in this case, the identity at 5′ and 3′ ends dropped to 71.9% and 63.5%, respectively (supplementary table S2, Supplementary Material online).

The central variable region observed in *Persaeus* elements has no obvious similarity with respective LINEs, the nt fragments being scattered across the length of Zenon ORF with only small stretch of local similarity (supplementary dataset S1, Supplementary Material online).

**Fig. 3.**
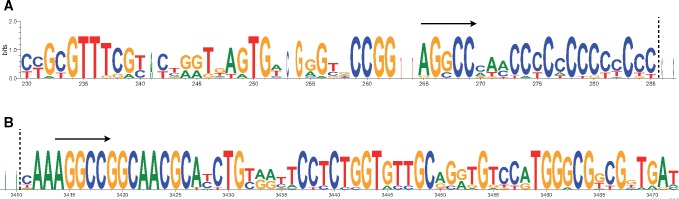
—Sequence logo of *Zenon* region surrounding the break point where internal deletions occur (dashed, vertical lines). (*A*) The region upstream the break point. (*B*) The region downstream the break point. Black arrows in (*A*) and in (*B*) indicate direct repeats (microhomologies).

The 5′ end homologous region between *Zenon* and *Persaeus* terminates with a poly-(C) stretch (fig. 3) and it appears variable at break point among different SIDEs (fig. 1; supplementary dataset S1, Supplementary Material online); moreover, the *Zenon’s* region where internal deletion occurs is surrounded by 5 bp direct repeat 5′-AGGCC-3′ (fig. 3).

**Fig. 4.**
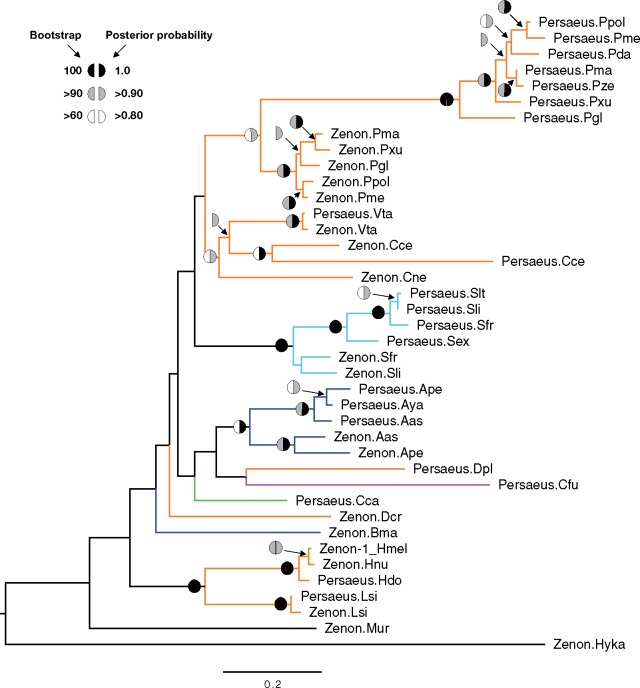
—Phylogenetic analyses of *Persaeus* and *Zenon* elements. Symbols at nodes represent support based on maximum likelihood bootstrap/Bayesian posterior probability, as reported on the upper left legend. Each element has been labelled by a suffix indicating the pertaining species: Aya, *Antheraea yamamai*; Ape, *A. pernyi*; Aas, *A. assama*; Bma, *Bombyx mandarina*; Cca, *Cadra cautella*; Cce, *Calycopis cecrops*; Cne, *Calephelis nemesis*; Cfu, *Choristoneura fumiferana*; Dcr, *Danaus chrysippus*; Dpl, *D. plexippus*; Hnu, *Heliconius numata*; Hdo, *H. doris*; Lsi, *Leptidea sinapis*; Hka, *Hyposmocoma kahamanoa*; Mur, *Megathymus ursus*; Pda, *Papilio dardanus*; Pgl, *P. glaucus*; Pma, *P. machaon*; Pme, *P. memnon*; Ppo, *P. polytes*; Pxu, *P. xuthus*; Pze, *P. zelicaon*; Sex, *Spodoptera exigua*; Sfr, *S. frugiperda*; Slt, *S. littoralis*; Sli, *S. litura*; Vta, *Vanessa tameamea*. Branch color codes as in figure 2.

In order to determine the evolutionary relationship between *Persaeus* and *Zenon*, phylogenetic analyses were carried out based on *Persaeus* and *Zenon* consensus sequences (supplementary dataset S1, Supplementary Material online) using maximum likelihood and Bayesian inference. The two phylogenetic analyses are fully congruent and indicated a clustering pattern of *Persaeus* and *Zenon* not based on the host Lepidoptera superfamilies but based on host genus or species (*Antheraea*, *Calycopis*, *Heliconius*, *Leptidea*, *Papilio*, *Spodoptera*, and *Vanessa*). Only *Persaeus* and *Zenon* from *Danaus* spp. resulted more distantly related (fig. 4). *Zenon* and *Persaeus* from the genera represented by more than one species (*Antheraea*, *Heliconius*, *Papilio*, and *Sopodoptera*) not only cluster in monophyletic clades but each of these clades shows two further subclades, one for *Zenon* and one for *Persaeus*. Notably, the *Persaeus* subclades showed a topology that appears generally congruent with the species phylogeny (supplementary fig. S5, Supplementary Material online).

**Fig. 5.**
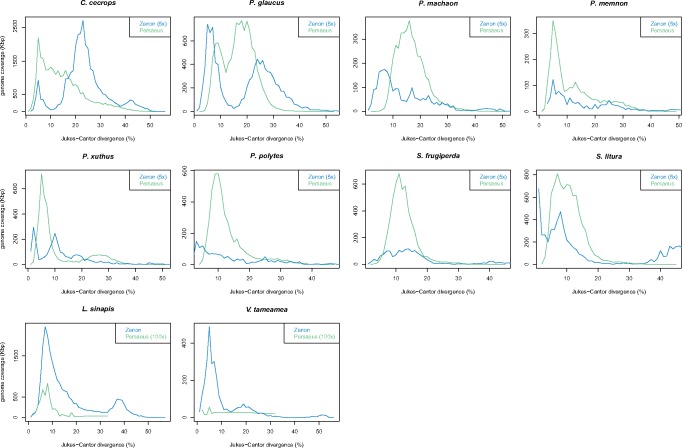
—*Persaeus* and *Zenon* activity through age analysis. Where necessary, data were magnified (as indicated in graph insets) in order to improve the readability.

When looking to activity through age analysis of *Persaeus* and *Zenon* in the same genome, we found an increase of *Persaeus* activity corresponding to the increased *Zenon* activity (fig. 5).

## Discussion

In this study, we identified a novel nonautonomous retroelement, the SIDE *Persaeus*, and analyzed the evolutionary dynamics with its partner CR1 LINE, *Zenon*, in Lepidoptera genomes. We also confirmed that CR1 retrotransposons, which are considered among the most abundant superfamily of TEs in the amniote genomes, are also abundant in insects, at least in Lepidoptera. Previous analyses already identified elements of the CR1 clade in insects, including Lepidoptera (Biedler and Tu 2003; Kapitonov and Jurka 2003; Novikova et al. 2007; Thompson et al. 2009). In the present analysis, we characterize the full-length sequence of additional 18 *Zenon* CR1 elements in further lepidopteran species. Most of LINEs, and especially CR1 elements, are frequently truncated at the 5′ end (Hillier et al. 2004), which make difficult to reconstruct the full-length CR1 as well as determine the exact boundary of their 5′ end. This, in part, explains why in some genomes we cannot retrieve full-length *Zenon* elements.

The finding and the evolutionary dynamics of the retrieved SIDE, *Persaeus*, are remarkable because this is, to our knowledge, the first instance of several independent successful genome invasions by an SIDE. Other SIDEs, such as R2 SIDE and R2/R1 hybrid SIDEs (Eickbush and Eickbush 2012), Ag-Sponge (Biedler and Tu 2003), and TbRIME (Kimmel et al. 1987) had been reported, but almost all these SIDEs as well as partner LINEs had low copy number in their host genomes. This has been attributed to various factors, among which the ability of the SIDE to be transcribed into RNA (Eickbush and Eickbush 2012). The other known successful nonautonomous retroelements, SINEs, are transcribed by the RNA pol-III thanks to the presence of promoter sequences in the RNA-related head (Luchetti and Mantovani 2013). The sequence of *Persaeus* 5′ end includes part of the CR1 5′-UTR, showing between 71% and 98% of identity, which is important for transcription because, in LINEs, it contains the promoter sequence (Lee et al. 2012). Therefore, *Persaeus* could retain the potential to be transcribed by the same mechanism of its partner *Zenon*. It has been showed that the 3′-UTRs of LINEs, including CR1 elements, is used as a recognition site for the encoded RT (Kajikawa et al. 1997; Haas et al. 2001; Suh 2015). Like the functional relationship between partner SINEs and LINEs, that is mediated by the similar nt sequence at the 3′ end (Ohshima and Okada 2005), *Persaeus* exhibited a 3′ end sharing 72–99% of identity with the *Zenon* 3′-UTR: Therefore, this suggests that it might borrow the retrotransposition machinery from its autonomous partner *Zenon*. This is also supported by the activity through age analysis, where *Persaeus* activity resulted contemporary to that of the partner *Zenon* in all assayed genomes.

Overall, at variance of previously identified SIDEs, it appears that *Persaeus* underwent to a replicative burst during Lepidoptera evolution reaching, on average, the 1.48% in length of the host genomes, with the remarkable instance of *C. cecrops* whose genome is made by the 3.48% of *Persaeus* SIDEs. *Zenon* activity showed the same trend, reaching an average genome coverage of 0.75% and with the maximum value scored in *L. sinapis* (3.45%).

Although the general structure of *Persaeus* is conserved across species, the sequences comparison revealed a more complex pattern. First of all, the regions homologous to *Zenon* 5′ and 3′ ends showed different structures that are consistent among closely related species (e.g., like the congeneric ones) but well differentiated between distantly related taxa. Moreover, the alignment pattern of the central variable region, which do not show any clear relationships with *Zenon* or any other sequences, suggest a nonhomologous origin. The phylogenetic analysis performed on SIDEs and LINEs indicated a concordant pattern of evolution. In fact, *Persaeus* and *Zenon* elements isolated from species of the same genus always cluster together, forming an SIDE and an LINE subclade in each genus or species clade (*Antheraea*, *Calycopis*, *Heliconius*, *Lepitdea*, *Papilio*, *Spodoptera*, and *Vanessa*). Altogether, the nonhomologous sequence structure and the phylogenetic pattern suggests that, although widespread among lepidopteran, the emergence of *Persaeus* occurred multiple time by internal deletion of a clade-specific *Zenon* element. The alternative hypothesis of a single origin of *Persaeus* appears unlikely as, in that case, we would have observed in the phylogenetic tree a single, ancient split between *Zenon* and *Perseus* sequences and then their diversification in the different clades. This is, actually, exactly the pattern that can be observed within those clades where multiple congeneric species are present (i.e., *Antheraea*, *Heliconius*, *Papilio*, and *Spodoptera*; fig. 4), suggesting that *Persaeus* emerged by *Zenon* internal deletion early during the evolution of these clades and that, because then, the two elements diverged independently. Moreover, when looking at the branching pattern within the *Antheraea, Papilio*, and *Spodoptera* clades it appeared that the *Persaeus* phylogeny resulted generally similar that of the host species. Although the taxon sampling is not exhaustive, as it is limited to genomic/transcriptomic data available for these genera in the database, this would suggest that the SIDE emerged in the common ancestor of each genus and then it was inherited following a vertical pattern. TEs are able to be transmitted by horizontal transfer, although with different rates based on specific biological feature of the element itself and of the host organism (Scavariello et al. 2017). Recent surveys on insect TEs indicated a global high frequency of horizontal transfers, evidencing a particular tendency of Lepidoptera to be involved in these events (Peccoud et al. 2017; Reiss et al. 2019). Moreover, it was found that these events preferentially took place among closely related species (Peccoud et al. 2017). However, horizontal transfers of nonautonomous retrotransposons have been only rarely reported (Hamada et al. 1997; Piskurek and Okada 2007; Luchetti et al. 2016; Luchetti and Mantovani 2016): This is probably because the lack of the specific partner autonomous element in the landing genome do not allow the replication of the transferred element. In the case of *Persaeus*, though, the co-occurrence of similar active LINEs could make possible such hypothetical successful horizontal transfer. Our data, apparently, seem to rule out this possibility in the assayed genomes but the *Persaeus*/*Zenon* partnership identified in this study might also provide an ideal system to investigate these interactions.

**Fig. 6.**
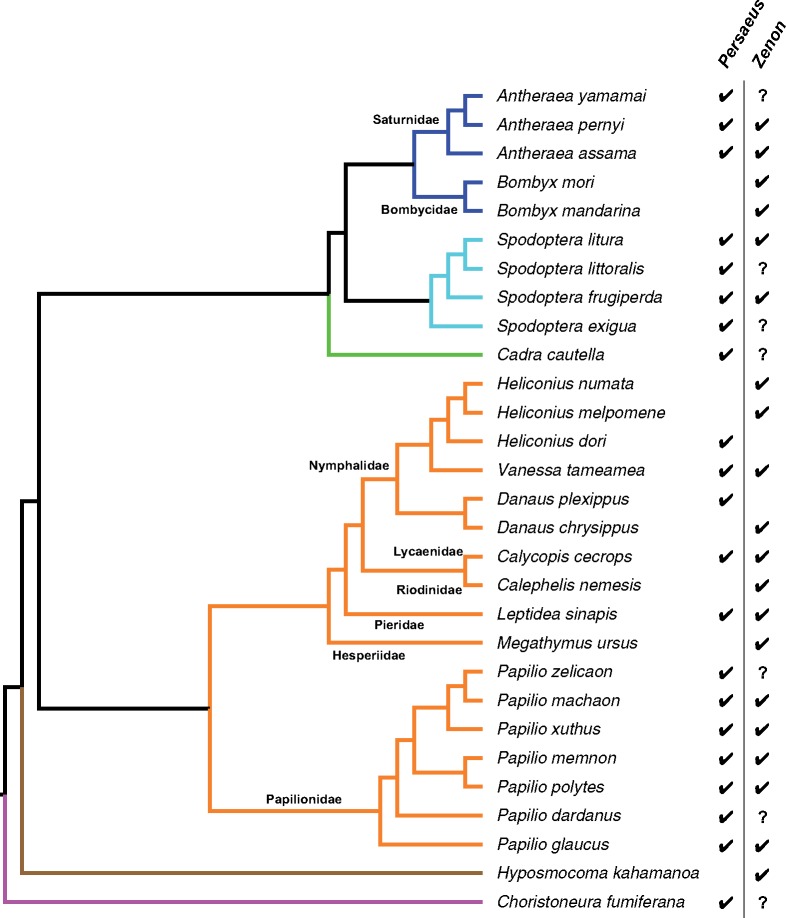
—Summary of *Persaeus* and *Zenon* distribution across the hosts phylogeny. Question marks indicate species where genome sequence was not available and, therefore, the absence of *Zenon* maybe to the limited data set available (GenBank database EST, TSA, or nt; table 1).

Although tested on a potentially limited taxon sampling, looking at the differential distribution of *Persaeus* and *Zenon* on the phylogeny of presently analyzed species (fig. 6), it appears that in some lineages the SIDE did not emerged or do not raised to a detectable copy number. However, the fact that *Persaeus* originated multiple times, even if with slightly different structure and from different member of the CR1 *Zenon* subfamily, indicates the presence some structural motif that may facilitate the internal deletion. *Zenon* sequence inspection evidenced the presence of short direct repeats bordering the region where internal deletion occurred. The presence of short direct repeats, also called microhomologies, has been thought to promote internal deletions among class II TE, through a DNA repair mechanism triggered after element excision (Rubin and Levy, 1997; Negoua et al., 2013). However, *Zenon* is a class I element where excisions, although possible, are rare events (van de Lagemaat et al. 2005). Another possible explanation for the frequent emergence of internal deletion derivatives could rely on recombination: Microhomologies could serve as nonhomologous sequences pairing region and recombination may occur. Interestingly, this could happen also during the reverse transcription process, as described in the copy-choice RNA recombination model: The RT enzyme is able to switch RNA template (template jump) between region of sequence similarity, leading to chimeric molecules (Simon-Loriere and Holmes 2011). This model has been repeatedly reported as potential generator of new SINE elements (Szafranski et al. 2004; Luchetti and Mantovani 2016). Moreover, the RT enzyme could be able to add nontemplate nt while template jumping (Bibillo and Eickbush 2004): This could possibly explain the presence of nt stretches in the central variable region of *Persaeus* which are not clearly related to other elements’ sequences.

Overall, the evolutionary consequences of the amplification burst of *Persaeus* and *Zenon* found here need to be further investigated, even because the invasion of substantial fraction of DNA generated by transposition of TEs can strongly affect the structure and functionality of genomes (Feschotte and Pritham 2007; Cordaux and Batzer 2009). CR1 transposons are widespread in the genomes of amniotes and they were the only active transposons during the avian lineage evolution (Hillier et al. 2004; Kaiser et al. 2006; Warren et al. 2010; Haddrath and Baker 2012; Liu et al. 2012; Baker et al. 2014). The characteristics of widespread distribution and high copy number of *Persaeus* and *Zenon* seem to imply that CR1 elements are also active throughout Lepidoptera evolution.

## Supplementary Materials

Supplementary data are available at *Genome Biology and Evolution* online. 

## Data Availability

All data generated or analyzed during this study are included in this published article and its supplementary information files.

## Ethics Approval

Not applicable.

## Funding

This work was supported by the Funds for Distinguished Young Scientists of Jiangxi Province (20192BCBL23028), the National Natural Science Foundation of China (31700318 and 31560308) to H.H.Z and by Canziani funding to A.L.

## Authors’ Contributions

H.H.Z., P.L.W., and A.L. designed and supervised the study. A.L., A.A.R., P.L.W., M.R.X.X., X.M.X., X.G.Z., and H.H.Z. performed bioinformatic analyses. P.L.W., X.M.X., X.G.Z., H.H.Z., and A.L. wrote and revised the manuscript.

## Supplementary Material

evz174_Supplementary_Data
