# Supplementary material for: Successful Invasions of Short Internally Deleted Elements (SIDEs) and Its Partner CR1 in Lepidoptera Insects
Source: Genome Biol Evol. 2019 Aug 6;11(9):2505–16. doi: 10.1093/gbe/evz174 (PMC6740152; doi:10.1093/gbe/evz174)
Supplement: evz174_Supplementary_Data [file evz174_supplementary_data.zip › Figure S2.pdf]

```
Garfield_SL   : GAGCCGATTGTTGAAGCGGAAAAAGTATTAATGCTGCCTTTGCACATTAAGTTAGGGTTGATGAAACAATTTGT----TAAAAAACTGGATGAAACTTCAGAAGCTTTTGGATA : 110
Garfield_SC   : GAGCCGATTGTTGAAGCGGAAAAAGTGTTAATGCCGCCTTTGCACATTAAGTTAGGGTTGATGAAACAATTTGT----TAAAAAACTGGATGAAACTTCAGAAGCTTTTGTATA : 110
Garfield_BM   : GAGCCGATTGTTGAAGCGGAAAAAGTGTTAATGCCGCCTTTGCACATTAAGTTAGGGTTGATGAAACAATTTGT----TAAAAAACTGGATGAAACTTCAGAAGCTTTTGGATA : 110
Garfield_RP   : GAGCCGATTGTTGAAGCGGAAAAAGTGTTAATGCCGCCTTTGCACATTAAGTTAGGGTTGATGAAACAATTTGT----TAAAAAACTGGATGAAACTTCAGAAGCTTTTGGATA : 110
Garfield_AP.1 : GAGCCGA-TGTTGAAGCGGAAAAAGTGTTA-------------ACATTAAGTTAGGGTTGATGAAACAATTTGTTAAATAAAAAATTGGATGAAAATTCAGAAGCTTTTAGATA : 100
Garfield_AP.2 : GAGCCGATTGTTGAAGCGGAAAAAGTGTTA-------------ACATTAAGTTAGGGTTGATGAAACAATTTGTTAAATAAAAAATTGGATGAAAATTCAGAAGCTTTTAGATA : 101

Garfield_SL   : CTTAAA-TTTTTTTTTCCCAAGTTATCGGAAGCAAAG--------------TTTTGTTGGTCCGCAAATAAGACAGATTTTCGCCGATGAAAAATTTCCAACGT--TCCTGAAT : 207
Garfield_SC   : CTTAAAAATTTTTTTTCCGAAGTTATCGCAAGCAAAG--------------TTTTGTTGGTCCGCAAATAAGACAGATTTTCGCCGATGAAAAATTTCCAACGT--TGCTGAAT : 208
Garfield_BM   : CTTAAAAAAATTTTTTCCGAAGTTATCGGAAGCAAAGGTTAAAGCTGGGGTTTTTGTTGGTCCGCAAATAAGACAGATTTTCGCCGATGAAAAATTTCCAACGT--TGCTGAAT : 222
Garfield_RP   : CTTAAAAAAATTTTTTCCGAAGTTATCGGAAGCAAAGGTTAAAGCTGGGGTTTTTGTTGGTCCGCAAATAAGACAGATTTTCGCCGATGAAAAATTTCCAACGT--TGCTGAAT : 222
Garfield_AP.1 : CTTGAACAAGATTTTTCCAAAGTTATCAGAAGCAAAAGTTAAAGCTGGGGTTTTTGTTGGTCCACAAACAA-----ATTTTCGAAGATGAAAAATTTCTTATGTTGTGCTGAAT : 209
Garfield_AP.2 : CTTGAACAAGATTTTTCCAAAGTTATCAGAAGCAAAAGTTAAAGCTGGGGTTTTTGTTGGTCCACAAACAA-----ATTTTCGAAGATGAAAAATTTCTTATGTTGTGCTGAAT : 210

Garfield_SL   : CGTACTCAAAAAGCAAGTTGGAACAGTTTTAAAGCAGTAGTTTCTGGA------------------------------------------------------------------ : -
Garfield_SC   : CGTACTCAAAAAGCAAGTTGGAACAGTTTTAAAGCAGTAGTTTCTGGA------------------------------------------------------------------ : -
Garfield_BM   : CGTACTCAAAAAGCAAGTTGGAACAGTTTTAAAGCAGTAGTTTCTGGA------------------------------------------------------------------ : -
Garfield_RP   : CGTACTCAAAAAGCAAGTTGGAACAGTTTTAAAGCAGTAGTTTCTGGA------------------------------------------------------------------ : -
Garfield_AP.1 : CGTGCTC-AAAAGCGAGTTGGAACAGTTTCAAATCAGTAGTTTCTGAATTTTTTCATTTTCATCACATTTCTTGATGGTGGTAGAGCCACGCTGAAACTATTACTGGTTTCAAC : 322
Garfield_AP.2 : CGTGCTC-AAAAGCGAGTTGGAACAGTTTCAAATCAGTAGTTTCTGAATTTTTTCATTTTCATCACATTTCTTGATGGTGGTAGAGCCACGCTGAAACTATTACTGGTTTCAAC : 323

Garfield_SL   : ------------------------------------------------------------------------------------------------------------------ : -
Garfield_SC   : ------------------------------------------------------------------------------------------------------------------ : -
Garfield_BM   : ------------------------------------------------------------------------------------------------------------------ : -
Garfield_RP   : ------------------------------------------------------------------------------------------------------------------ : -
Garfield_AP.1 : ATGAATGGGTCGGCTCGCCCGGGGTAGTACAACCCTCTCATAGAAAACCGACGTGAAGTAACTGGGCTTGCATTGTTGCGTTTCGTTCGGTCAGCGAGAGCGCCGGAGGCCCAC : 436
Garfield_AP.2 : ATGAATGGGTCGGCTCGCCCGGGGTAGTACAACCCTCTCATAGAAAACCGACGTGAAGTAACTGGGCTTGCATTGTTGCGTTTCGTTCGGTCAGTGAGAGCGCCGGAGGCCCAC : 437

Garfield_SL   : ------------------------------------------------------------------------------------------------------------------ : -
Garfield_SC   : ------------------------------------------------------------------------------------------------------------------ : -
Garfield_BM   : ------------------------------------------------------------------------------------------------------------------ : -
Garfield_RP   : ------------------------------------------------------------------------------------------------------------------ : -
Garfield_AP.1 : CTCCCCTCCCCTCTCTTCTCTCCTCTCCCTCTCCCTCCTCTAACCCTTCCCCCAAAGGGTGGCAACGCACTTGCGACTCCTCGGGTGTTGTTAGTGTCCATGGACGGTGGTGAT : 550
Garfield_AP.2 : CTCCCCTCCCCTCTCTTCTCTCCTCTCCCTCTCCCTCCTCTAACCCTTCCCCCAAAGGGTGGCAACGCACTTGCGACTCCTCGGGTGTTGTTAGTGTCCATGGACGGTGGTGAT : 551

Garfield_SL   : ----------------------------------------------------------TTTTTAGGAAATAATAAAGCTAAAAACTACGAAAAGTTGGTTGAGGATATGCTTAC : 311
Garfield_SC   : ----------------------------------------------------------TTTTTAGGAAATAATAAAGCTGAAAACTACGAAAAGTTGGTTGAGGATATGCTTAC : 312
Garfield_BM   : ----------------------------------------------------------TTTTTAGGAAATAATAAAGCTGAAAACTACGAAAAGTTGGTTGAGGATATGCTTAC : 326
Garfield_RP   : ----------------------------------------------------------TTTTTAGGAAATAATAAAGCTGAAAACTACGAAAAGTTGGTTGAGGATATGCTTAC : 326
Garfield_AP.1 : CACTTAACAGCAGGTGACCCATCTCTTCGTTTGCCACCTATGACATAAAAAAAAAATTTTTTTAGGAAATAATAAAGCTGAAAACTACAAAACGTTAGTTGAAGATATGCTAAA : 664
Garfield_AP.2 : CACTTAACAGCAGGTGACCCATCTCTTCGTTTGCCACCTATGACATAAAAAAAAAATTTTTTTAGGAAATAATAAAGCTGAAAACTACAAAACGTTAGTTGAAGATATGCTAAA : 665

Garfield_SL   : AAATTTTAAGGCCATGGGCTGCAGGATGTCATTAAAAGTACATATGCTGCATGCTCATTTGGATAA--A-TTTAAAAAC-ATATGGGAGCCTATTCCGAAGAGCAAGGAGAACG : 421
Garfield_SC   : AAATTTTAAGGCCATGGGCTGCAGGATGTCATTAAAAGTATATATGCTGCATGCTCATTTGGATAA--T-TTTAAAAACAATATGGGAGCCTATTCCGAAGAGCAAGGAGAACG : 423
Garfield_BM   : AAATTTTAAGGCCATGGGCTGCAGGATGTCATTAAAAGTACATATGCTGCATGCTCATTTGGATAA--A-TTTAAAAACAATATGGGAGCCTATTCCGAAGAGCAAGGAGAACG : 437
Garfield_RP   : AAATTTTAAGGCCATGGGCTGCAGGATGTCATTAAAAGTACATATGCTGCATGCTCATTTGGATAA--A-TTTAAAAACAATATGGGAGCCTATTCCGAAGAGCAAGGAGAACG : 437
Garfield_AP.1 : AAAAATTAAGACCATGATCTGCAGGATGTCATTAAAAGTACACATGCTGCATGCTCATTTAGATAATTAATTTAACAACAAGGTGGGAGCGGATTCAGAAGAGT------AGCA : 772
Garfield_AP.2 : AAAAATTAAGACCATGATCTGCAGGATGTCATTAAAAGTACACATGCTGCATGCTCATTTAGATAATTAATTTAACAACAAGATGGGAGCGGATTCAGAAGAGT------AACA : 773

Garfield_SL   : TTTCCATCAGGACATCATGAATTTTGAACAACGCTATCAAGGCCA : 466
Garfield_SC   : TTTCCATCAGGACATCATGAATTTTGAACAACGCTATCAAGGCCA : 468
Garfield_BM   : TTTCCATCAGGACATCATGAATTTTGAACAACGCTATCAAGGCCA : 482
Garfield_RP   : TTTCCATCAGGACATCATGAATTTTGAACAACGCTATCAAGGCCA : 482
Garfield_AP.1 : TTTTATTCAGGACATTATGAGCTTCGAACAACGCTATCAAGGCCA : 817
Garfield_AP.2 : TTTTATTCAGGACATTATGAGCTTCGAACAACGCTATCAAGGCCA : 818
```
