# Supplementary material for: Successful Invasions of Short Internally Deleted Elements (SIDEs) and Its Partner CR1 in Lepidoptera Insects
Source: Genome Biol Evol. 2019 Aug 6;11(9):2505–16. doi: 10.1093/gbe/evz174 (PMC6740152; doi:10.1093/gbe/evz174)
Supplement: evz174_Supplementary_Data [file evz174_supplementary_data.zip › Table S1.docx]

**Table S1**. Genome occupancy of *Persaeus* and *Zenon*.

| **Species** | **Taxonomy (Superfamily)** | ***Persaeus* bp** | ***% genome*** | ***Zenon* bp** | ***% genome*** |
| --- | --- | --- | --- | --- | --- |
| *Bombyx mandarina* | Bombycoidea |  |  | 2548635 | 0.64 |
| *Calephelis nemesis* | Papilionoidea |  |  | 3859958 | 0.48 |
| *Calycopis cecrops* | Papilionoidea | 26814577 | 3.68 | 6123237 | 0.84 |
| *Danaus chrysippus* | Papilionoidea |  |  | 157726 | 0.05 |
| *Danaus plexippus* | Papilionoidea | 832216 | 0.33 |  |  |
| *Heliconius melpomene* | Papilionoidea |  |  | 690011 | 0.25 |
| *H. numata* | Papilionoidea |  |  | 311215 | 0.09 |
| *H. doris* | Papilionoidea | 191329 | 0.07 |  |  |
| *Hyposmocoma kahamanoa* | Gelechioidea |  |  | 4398596 | 0.60 |
| *Leptidea sinapis* | Papilionoidea | 44317 | 0.01 | 22154437 | 3.45 |
| *Megathymus ursus* | Hesperioidea |  |  | 4745215 | 1.11 |
| *P. glaucus* | Papilionoidea | 11373850 | 3.03 | 1931966 | 0.52 |
| *P. machaon* | Papilionoidea | 4325032 | 1.55 | 458206 | 0.16 |
| *P. memnon* | Papilionoidea | 2734100 | 1.25 | 218024 | 0.10 |
| *P. polytes* | Papilionoidea | 4479765 | 1.97 | 290688 | 0.13 |
| *P. xuthus* | Papilionoidea | 3934088 | 1.61 | 498555 | 0.20 |
| *S. frugiperda* | Noctuoidea | 5182469 | 1.45 | 342870 | 0.10 |
| *S. litura* | Noctuoidea | 8406306 | 1.92 | 1136688 | 0.26 |
| *Vanessa tameamea* | Papilionoidea | 2927 | 0.001 | 3040842 | 0.85 |
