# Supplementary material for: Successful Invasions of Short Internally Deleted Elements (SIDEs) and Its Partner CR1 in Lepidoptera Insects
Source: Genome Biol Evol. 2019 Aug 6;11(9):2505–16. doi: 10.1093/gbe/evz174 (PMC6740152; doi:10.1093/gbe/evz174)
Supplement: evz174_Supplementary_Data [file evz174_supplementary_data.zip › Table S2.docx]

**Table S2**. Percentage of similarity at homologous regions between *Persaeus* and *Zenon* pairs.

| **SIDE/LINE pair** | **5' end** | **3' end** |
| --- | --- | --- |
| Persaeus/Zenon.Aas | 81.5 | 80.5 |
| Persaeus/Zenon.Ape | 76.8 | 75.3 |
| Persaeus/Zenon.Cce | 71.5 | 72.6 |
| Persaeus.Dpl/Zenon.Dcr | 71.9 | 63.5 |
| Persaeus.Hdo/Zenon-1_Hmel | 96.5 | 94.2 |
| Persaeus.Hdo/Zenon.Hnu | 95.4 | 94.5 |
| Persaeus/Zenon.Lsi | 98.0 | 97.6 |
| Persaeus/Zenon.Pgl | 71.0 | 60.7 |
| Persaeus/Zenon.Pma | 73.3 | 61.6 |
| Persaeus/Zenon.Pme | 74.8 | 60.5 |
| Persaeus/Zenon.Ppol | 71.7 | 60.4 |
| Persaeus/Zenon.Pxu | 74.4 | 61.8 |
| Persaeus/Zenon.Sfr | 77.3 | 77.9 |
| Persaeus/Zenon.Sli | 81.6 | 78.3 |
| Persaeus/Zenon.Vta | 98.9 | 99.2 |
