# Supplementary figures and images for: Successful Invasions of Short Internally Deleted Elements (SIDEs) and Its Partner CR1 in Lepidoptera Insects
Source: Genome Biol Evol. 2019 Aug 6;11(9):2505–16. doi: 10.1093/gbe/evz174 (PMC6740152; doi:10.1093/gbe/evz174)

M 1 2 3 4 5 6 7 8 9

1000 bp ►  
750 bp ►  
500 bp ►

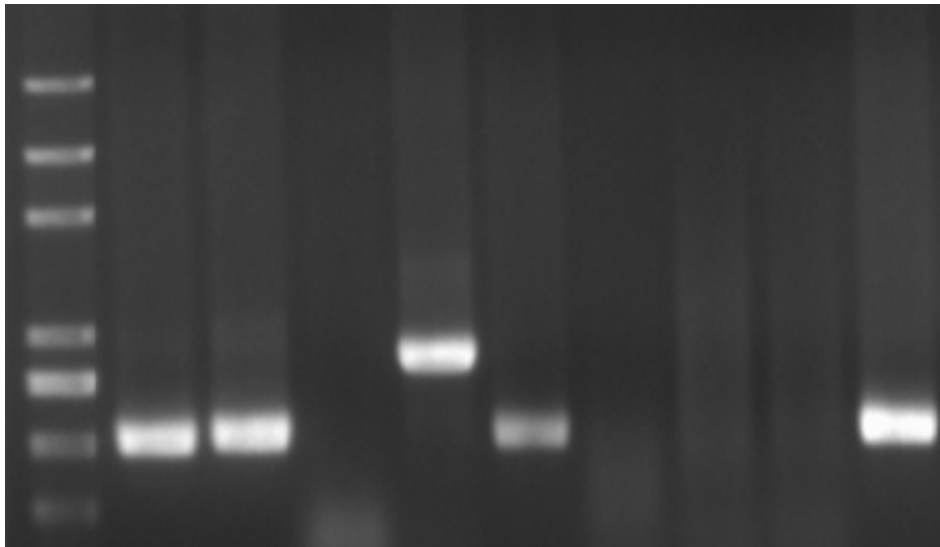

← 484 bp

Supplement: evz174_Supplementary_Data [file evz174_supplementary_data.zip › Figure S1.pdf]

A

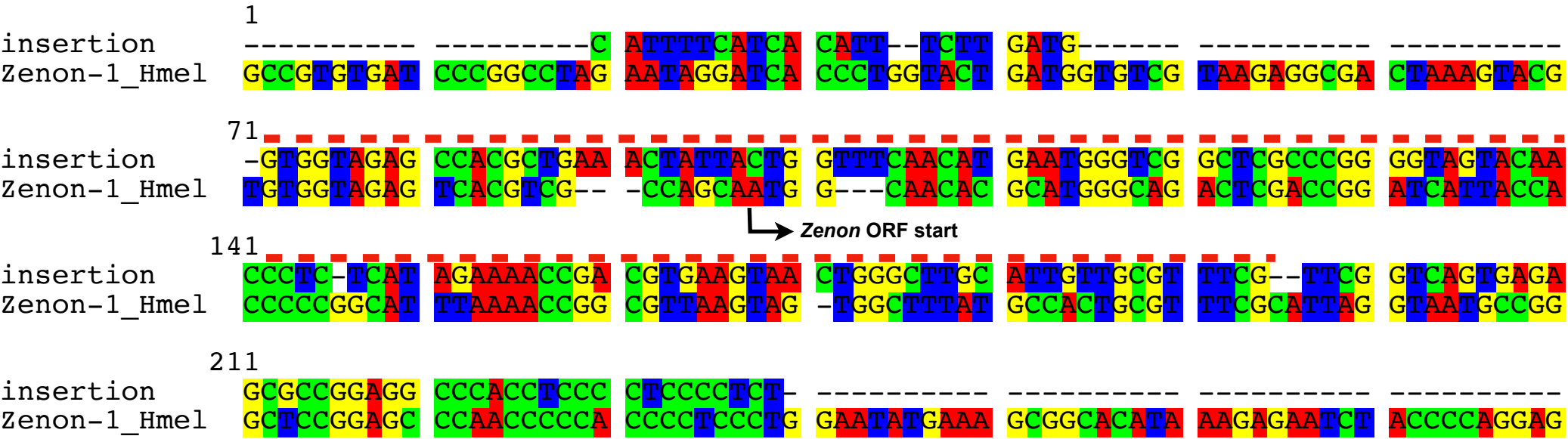

B

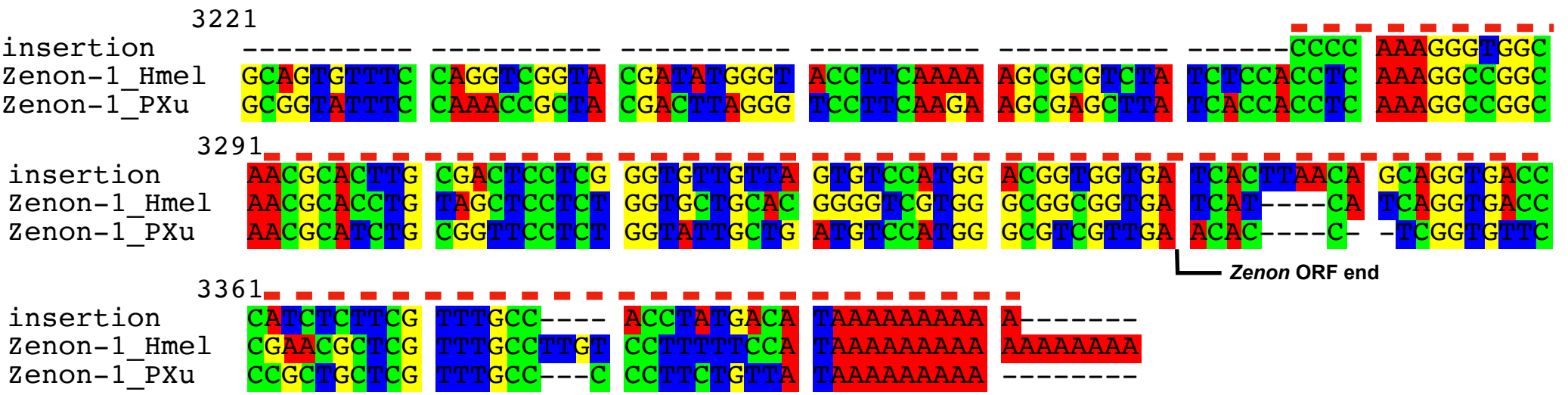

Supplement: evz174_Supplementary_Data [file evz174_supplementary_data.zip › Figure S3.pdf]

**Bootstrap**      **Posterior probability**

100   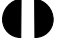   1.0

>90   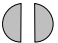   >0.90

>60   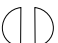   >0.80

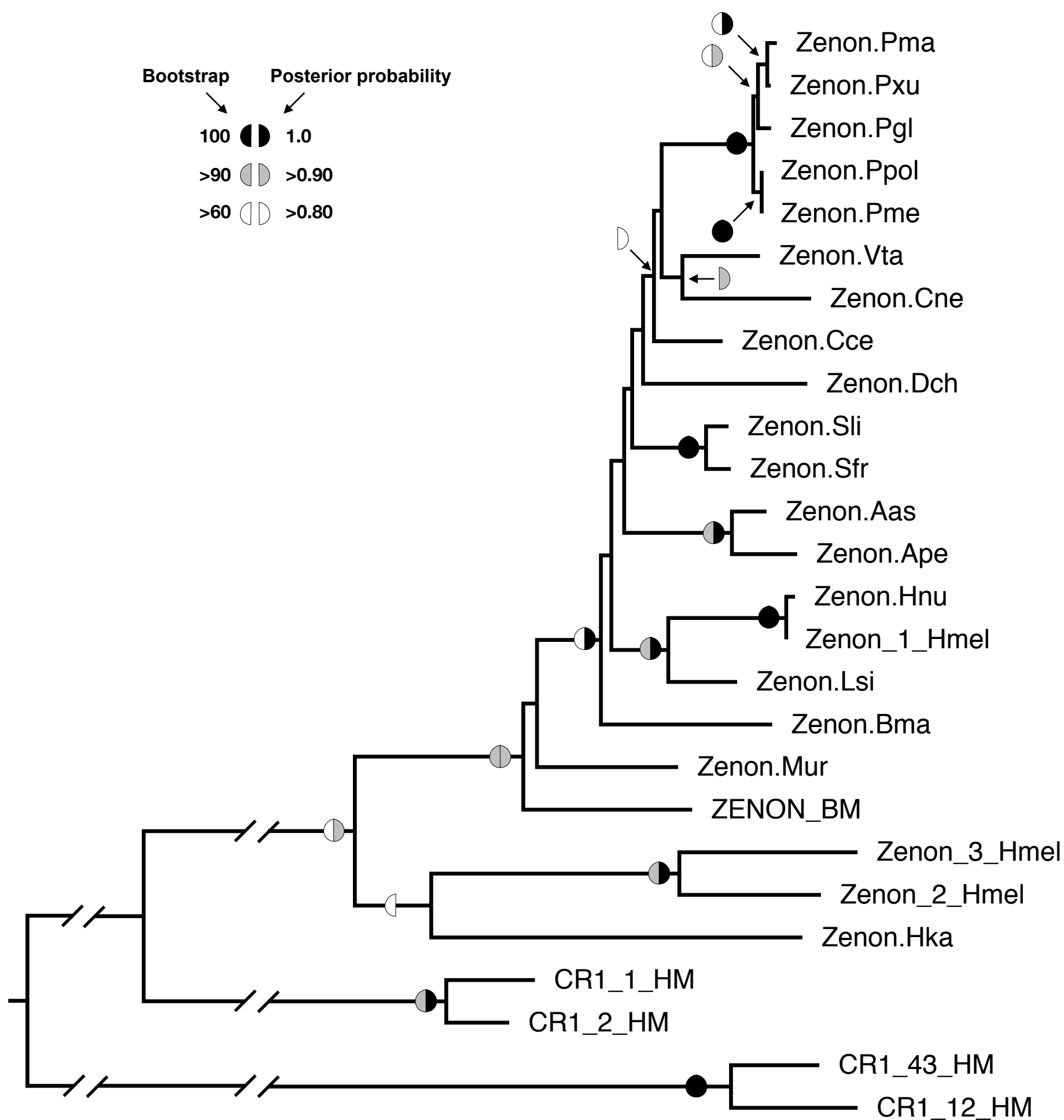

0.2

Supplement: evz174_Supplementary_Data [file evz174_supplementary_data.zip › Figure S4.pdf]

Bootstrap      Posterior probability

100 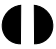 1.0  
 >90 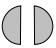 >0.90  
 >60 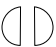 >0.80

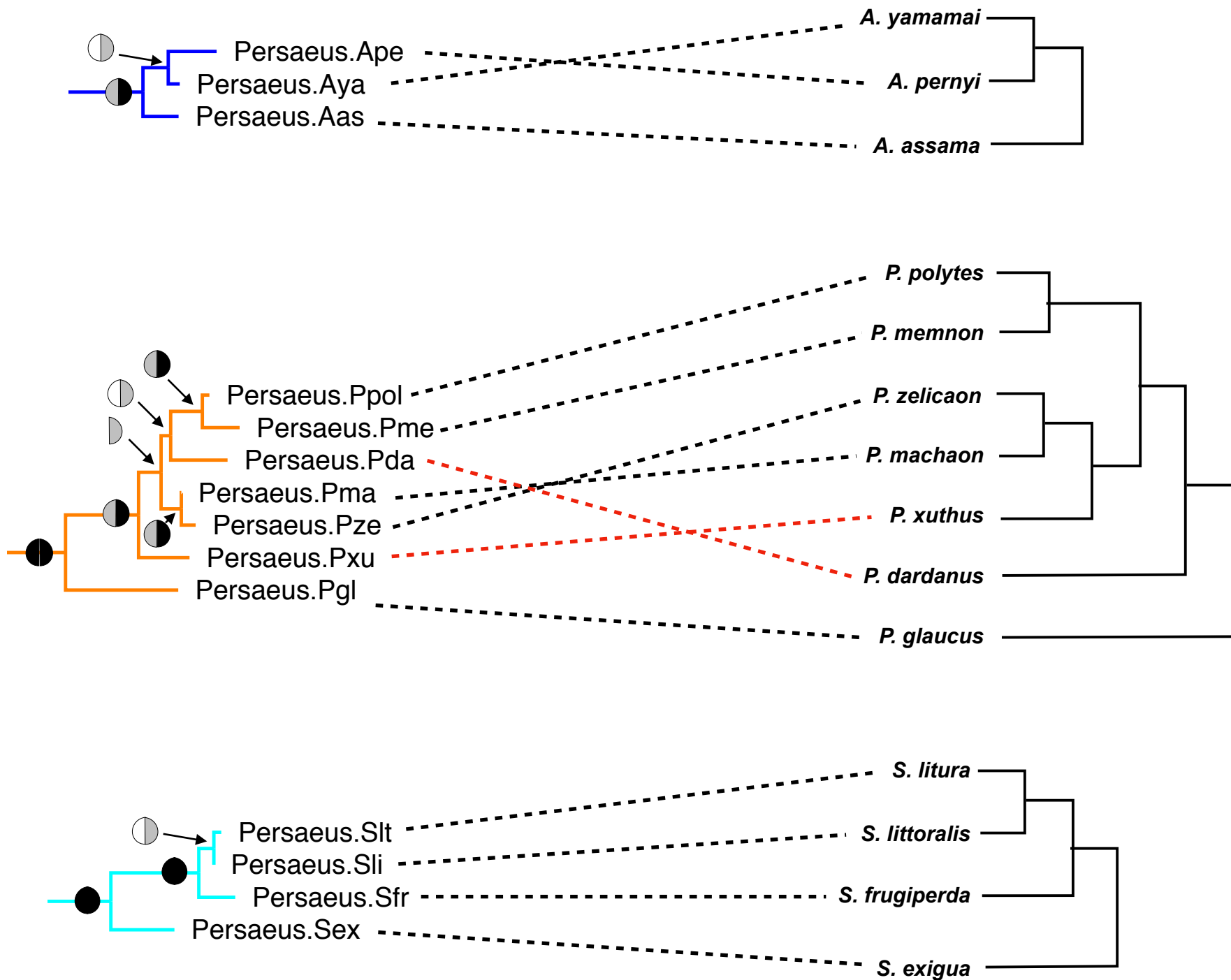

Supplement: evz174_Supplementary_Data [file evz174_supplementary_data.zip › Figure S5.pdf]
